# Metal-organic framework (MOF)-incorporated polymeric electrolyte realizing fast lithium-ion transportation with high Li^+^ transference number for solid-state batteries

**DOI:** 10.3389/fchem.2022.1013965

**Published:** 2022-10-03

**Authors:** Yifan Xu, Ruo Zhao, Jianjun Fang, Zibin Liang, Lei Gao, Juncao Bian, Jinlong Zhu, Yusheng Zhao

**Affiliations:** ^1^ Shenzhen Key Laboratory of Micro/Nano-Porous Functional Materials, Materials Science and Engineering, College of Engineering, Southern University of Science and Technology, Shenzhen, China; ^2^ Institute for Advanced Study Shenzhen University, Shenzhen, China; ^3^ Shenzhen Key Laboratory of Solid State Batteries, Guangdong Provincial Key Laboratory of Energy Materials for Electric Power, Academy for Advanced Interdisciplinary Studies, Southern University of Science and Technology, Shenzhen, China; ^4^ School of Materials Science and Engineering Peking University Beijing, Beijing, China

**Keywords:** metal-organic framework, composite polymer electrolyte, solid filler, ionic conductivity, pore confinement

## Abstract

Composite polymer electrolytes (CPEs) show significant advantages in developing solid-state batteries due to their high flexibility and easy processability. In CPEs, solid fillers play a considerable effect on electrochemical performances. Recently, metal-organic frameworks (MOFs) are emerging as new solid fillers and show great promise to regulate ion migration. Herein, by using a Co-based MOF, a high-performance CPE is initially prepared and studied. Benefiting from the sufficient interactions and pore confinement from MOF, the obtained CPE shows both high ionic conductivity and a high Li^+^ transference number (0.41). The MOF-incorporated CPE then enables a uniform Li deposition and stable interfacial condition. Accordingly, the as-assembled solid batteries demonstrate a high reversible capacity and good cycling performance. This work verifies the practicability of MOFs as solid fillers to produce advanced CPEs, presenting their promising prospect for practical application.

## 1 Introduction

As one of the paramount energy storage devices, Li-ion batteries (LIBs) have achieved great success as the power source for various portable electronics in our daily life over the past decades (Li et al., 2018; Duan et al., 2020). Nowadays, with the upsurge and development of electric vehicles, LIBs are facing new challenges, chiefly in terms of power density and safety. To fulfill these requirements, solid-state batteries (SSBs) using metallic Li anodes and solid-state electrolytes (SSEs) have triggered growing research interests (Krauskopf et al., 2020; Lou et al., 2020). In SSBs, Li anode with the low redox potential and high theoretical capacity offers great opportunities for harvesting intensified power. The SSEs without flammability and leakage problem could effectively eliminate the risk of catastrophic short circuits and correspondingly enhance operating safety (Liu et al., 2018; Cheng et al., 2019). Additionally, the physicochemical properties of SSEs are directly related to their electrochemical performance in SSBs (Cheng et al., 2019; Zhao Q. et al., 2020). Designing high-performance SSEs is of great significance to realize the real application of SSBs.

Generally, SSEs could be classified into two categories, including inorganic ceramics and composite polymer electrolytes (CPEs) (Fan et al., 2018). For inorganic ceramics (e.g., perovskite, garnet), they typically show high mechanical rigidity, which suffer from the problematic solid-solid interfacial contact with the electrodes (Gao et al., 2018; Lim et al., 2020). In contrast, CPEs that are comprised of polymer matrices, solid fillers, and Li salts, have high flexibility and are advantageous in building superior interfaces with reduced contact resistance (Yu et al., 2019; Zhu et al., 2019; Fan et al., 2020; Wang et al., 2020; Ye et al., 2020). Meanwhile, CPEs are now facing several challenges, such as the inferior mechanical strength, and the low ionic conductivity, which could be ascribed to the soft polymeric nature and insufficient internal interaction (Meyer, 1998). The introduction of solid fillers can be a way to optimize the CPEs’ properties. The interactions between the solid fillers, polymer matrices, and lithium salts can favor the salt dissociation and Li^+^ migration (Ye et al., 2020). Thus, a judicious selection and precise design of solid fillers are important to promote the electrochemical performance of the CPEs for battery utility.

Recently, metal-organic frameworks (MOFs) have become an emerging type of solid fillers with great application prospect in designing high-performance CPEs (Kumar et al., 2014; Wang et al., 2018b; Dutta and Kumar, 2019; Huo et al., 2019; Mathew et al., 2019; Miner and Dincă, 2019; Zhao R. et al., 2020). MOFs are crystalline porous materials that are assembled by metal ions/clusters and organic ligands. Compared with conventional inorganic fillers (e.g., SiO_2_, TiO_2_), MOFs show more fascinating features, including the high surface area, rich porosity, ordered channels, controllable structure and composition (Furukawa et al., 2010; Stock and Biswas, 2012; Zhou et al., 2012). These features endow MOFs great opportunities to manipulate the CPEs’ electrochemical performance and investigate the underlying structure-property relationship. Briefly speaking, the large surface area of MOFs can favor the contact and interaction with other components in CPEs, enhancing the densities of mobile Li^+^ and the conductive pathways (Gerbaldi et al., 2014). The ordered channels of MOFs could guide the Li plating/stripping processes and benefit the formation of stabilized interface during battery operation (Wang et al., 2018a; Wang et al., 2019). Despite these merits, there are still many challenges to be overcome for MOF-incorporated CPEs. In previous studies, the introduction of MOF into CPEs have been proven effective in improving ionic conductivity and mechanical strengh (Angulakshmi et al., 2014; Gerbaldi et al., 2014). However, How MOF particles affect ion migration is not clearly explained. Additionally, a detailed investigation of MOF’s intrinsic nature (e.g., pore shape, surface polarity) on battery performance needs to be clarified.

Herein, a cobalt-based MOF, ZIF-67, was synthesized and used as functional fillers to produce high-performance CPEs for SSBs. The benefits from MOF’s surface polarities and pore-confinement effect have been emphasized, which resulted in selective ion migration and an improved Li^+^ transference number. Specifically, ZIF-67 with high surface area enabled a sufficient contact with the lithium bis(trifluoromethanesulfonyl)imide (LiTFSI) and polyethylene oxide (PEO) matrix, and thus accelerate the salt dissociation and the segmental motion of polymer chains. The enhanced mobile Li^+^ and migration pathways then contribute to an improved ionic conductivity of 1.73 × 10^−4^ S cm^−1^ at 60°C. In addition, ZIF-67 had abundant cage-like micropores that could somewhat immobilize the long-columnar TFSI^−^ anions, thus improving the Li^+^ transference numbers. The ordered channels and rich porosity of ZIF-67 also contributed to a uniform Li^+^ reflux at the interface, which reduced the posibility of lithium dendrite formation and favored the long-term Li plating/stripping process. Consequently, the as-assembled Li symmetrical cell and full batteries demonstrate excellent performances. This work proves the functional role of MOFs as solid fillers in improving the performance of SSEs and their practical application in SSBs.

## 2 Results and discussion

### 2.1 Characterization of ZIF-67 filler and PLMs

**FIGURE 1:**
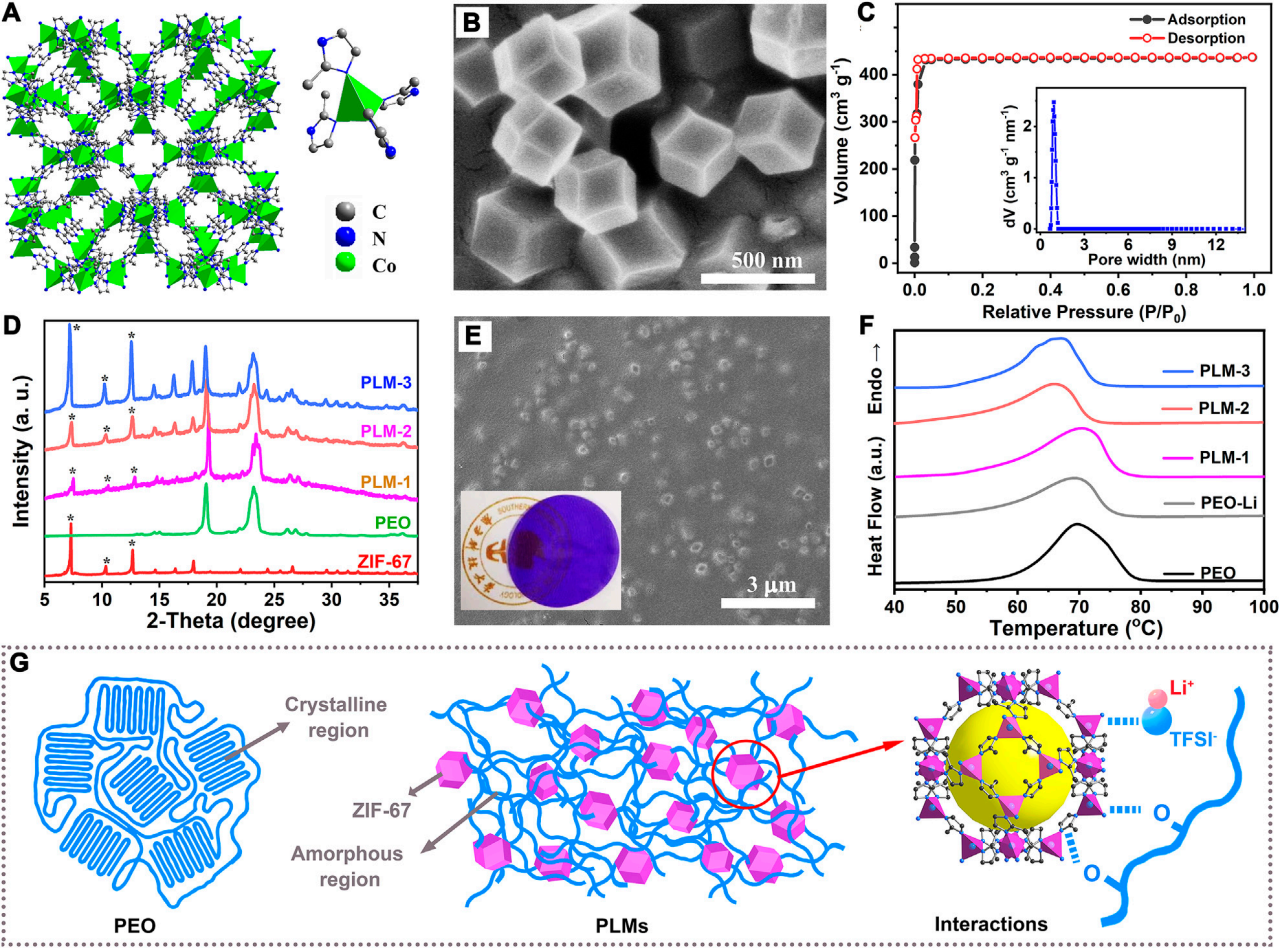
**(A)** The topology and coordination of ZIF-67. **(B)** The SEM image of ZIF-67. **(C)** The N_2_ adsorption curve and pore size distribution of ZIF-67. **(D)** The XRD patterns of as-synthesized ZIF-67, PLM-1, PLM-2 and PLM-3. **(E)** The SEM image of PLM-2. Inset is the photo of PLM-2 film. **(F)** The DSC curves of PEO, PEO-Li, PLM-1, PLM-2 and PLM-3. **(G)** Illustration of PLMs and the interactions.

The reaction of Co^2+^ and 2-methylimidazole produced a dodecahedron-shaped purple MOF (Co(C_4_N_2_H_5_)_2_, namely, ZIF-67). In ZIF-67, each Co^2+^ was coordinated by four nitrogen atoms from the 2-methylimidazolate anions periodically to construct porous frameworks and assembled into a sodalite topology (Figure 1A). The scanning electron microscopic (SEM) image (Figure 1B) showed that ZIF-67 nanoparticles possessed a dodecahedron-shaped morphology with a relatively uniform size distribution around 450 nm. Powder X-ray diffraction (PXRD) profile of the as-synthesized ZIF-67 matched well with the simulated diffraction patterns, which confirmed the successful synthesis of phase-pure ZIF-67 with high crystallinity (Supplementary Figure S1 in the Supporting information). The N_2_ sorption isotherms revealed that the ZIF-67 was microporous, with a Brunauer–Emmett–Teller surface area as high as 1,420 m^2^ g^−1^ (Figure 1C), and a microporous volume of 0.67 ml g^−1^. When used as solid fillers, ZIF-67 with such a high surface area and porosity could efficiently benefit the contact and interactions with the polymer matrix and lithium salts, and thus promote the ion diffusion.

With the increase of ZIF-67 content, three kinds of polyethylene oxide (PEO)-based CPEs (PLM-1, PLM-2 and PLM-3) were prepared. 0.1, 0.3 and 0.5 g ZIF-67 nanoparticles were added to 2.4 g PEO and 0.3 g LITFSI, named PLM-1,PLM-2 and PLM-3, respectively. The characteristic peaks for ZIF-67 could be clearly identified in the XRD patterns of PLM-1, PLM-2, and PLM-3, proving the electrochemical robustness of ZIF-67 in contact with PEO and LiTFSI (Figure 1D). In the XRD pattern of pure PEO, there were two obvious peaks at 19^o^ and 23^o^ that were assigned to the (1 2 0) and (1 1 2) crystal planes, respectively (Polu and Rhee, 2016). After the addition of ZIF-67, the relative intensities and broadness of these characteristic PEO peaks varied in some extent. These changes indicated that ZIF-67 could interact the PEO matrix and increase its amorphous degree. Additionally, the diffraction peaks for LiTFSI were absent in the XRD patterns of PLM-1, PLM-2, and PLM-3, indicating the complete dissolution of LiTFSI in PEO matrix and the provision of Li^+^ as charge carriers.

Figure 1E and Supplementary Figure S2 in the Supporting information depicted the surface morphologies of PLM-1, PLM-2, and PLM-3. All the PLMs showed a smooth surface, on which the distribution of MOF particles varied due to the different ZIF-67 contents. In PLM-1 and PLM-2, the ZIF-67 nanoparticle could be uniformly dispersed on the PEO matrix. The higher ZIF-67 content in PLM-2 over PLM-1 gave a much dense distribution. The further increase of ZIF-67 in PLM-3, however, led to the phenomenon of particle agglomeration. The thermal gravimetric analysis (TGA) was performed on PEO, PEO-Li, PLM-1, PLM-2, PLM-3, and ZIF-67 to measure their thermal stability, which is an important factor determining both the utilization conditions and the operational safety (Supplementary Figure S3 in the Supporting information). The TGA curve of PEO exhibited a sharp weight loss at around 330°C, while ZIF-67 showed high thermal stability with the decomposition temperature as high as 540°C. The residues of PLM-1, PLM-2, and PLM-3 took up approximately 6.1, 11.8, and 18.5 wt% at 450°C, much higher than that of pure PEO (1.5 wt%) due to the addition of ZIF-67 fillers.

The above analyses demonstrated that ZIF-67 possesses high surface area, rich porosity and excellent stability, which can be beneficial as functional solid fillers for CPEs. The merits of ZIF-67 could not only benefit the interaction with PEO and LiTFSI, but also alleviate the risk of thermal runaway in some degree.

The crystallinity of PEO matrix determines the segmental mobility of the polymer chains, which could affect the Li^+^ transfer process and thus the ionic conductivity of the CPEs. Differential scanning calorimetry (DSC) measurement was performed to evaluate the crystallinity degree (χ_c_) of PEO matrix and investigate the influence of ZIF-67 addition. As shown in Figure 1F, the DSC curves of PEO, PEO-Li, PLM-1, PLM-2, PLM-3 exhibited an obvious endothermic peak in the temperature range of 65–72^o^, which corresponded to the melting temperature (T_m_) of the PEO matrices. The measured T_m_ and the corresponding melting heat (ΔH_m_) were used to analyze the amorphous degree of PEO matrices. Upon the addition of ZIF-67, the T_m_ and ΔH_m_ showed noticeable changes. The pure PEO demonstrated the highest T_m_ of 70°C with the largest ΔH_m_ of 193.2 J g^−1^. Comparatively, PLM-2 with an addition of 10 wt% ZIF-67 possessed the lowest T_m_ and ΔH_m_ (66°C, 66.2 J g^−1^), reflecting the ease of mobility of PEO chains. The relative crystallinities (χ_c_) of PEO-Li, PLM-1, PLM-2, and PLM-3 were further estimated from the ratio of their corresponding ΔH_m_ to that of pure PEO, respectively. As shown in Supplementary Table S1, negative shifts of ΔH_m_ and χ_c_ were observed upon the introduction of LiTFSI and ZIF-67. The χ_c_ values for PLM-1, PLM-2, and PLM-3 were reduced to 65, 34, and 46%, respectively, which revealed the enhanced amorphous nature of PEO matrices.

The incorporated ZIF-67 with high surface area could effectively interact with PEO matrices, which interrupted the alignment of the PEO chains and thus decreased the crystallinity of the composite systems (Figure 1G). Benefiting from the accelerated mobility of PEO chains, the hopping sites for Li^+^ transport and the correspondingly ionic conductivity could be greatly improved. However, when the percentage of ZIF-67 was increased to 15.6 wt% for PLM-3, there was an unsatisfactory increase in crystallinity. This was presumably due to the agglomeration of ZIF-67 nanoparticles that attenuated the interactions with PEO chains. Thus it can be seen, the introduction of ZIF-67 with an optimal amount is an effective strategy in altering the motion behavior of PEO matrices, and could have a marked impact on the process of Li^+^ conduction. In addition, With the addition of MOF particles, the mechanical properties of the composite solid polymer electrolyte could be optimized. As shown in Supplementary Figure S10, The stress of PLM-2 was 8.5 MPa and the stain was 45%. With the increase of MOF contents, the stress was increased, while the strain was decreased. Compared with PLM-1 and PLM-3, PLM-2 showed a much better balance between the strength and strain, which enabled a better interfacial condition for batteries.

### 2.2 Electrochemical performances

**FIGURE 2:**
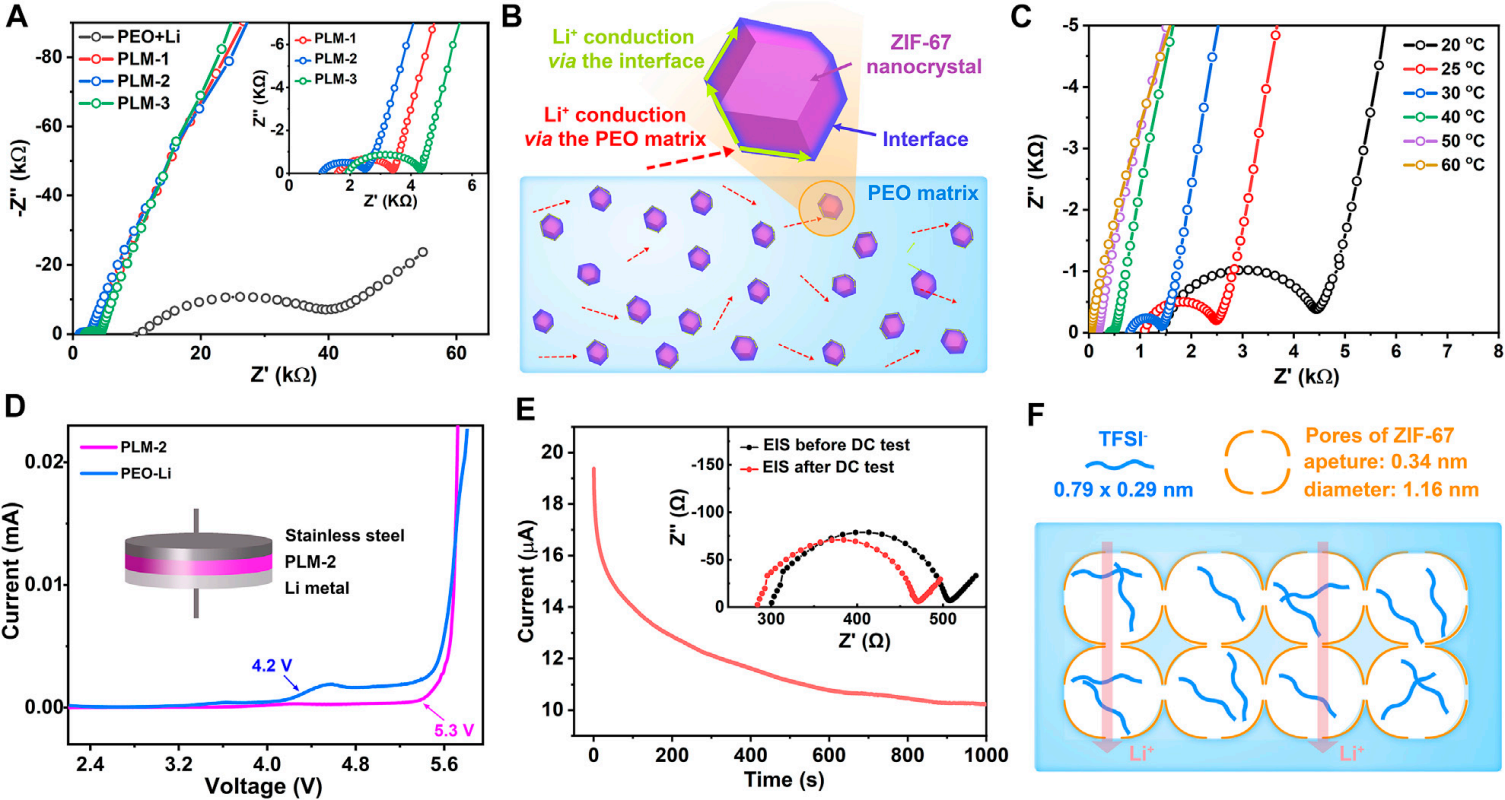
**(A)** The Nyquist plots of PEO-Li, PLM-1, PLM-2 and PLM-3. Inset is the enlargement curves at high frequencies. **(B)** Illustration of the different pathways for Li^+^ conduction. **(C)** The Nyquist plots of PLM-2 from 20 to 80°C. **(D)** The LSV curve of PLM-2 from 2 to 6 V at 60°C. **(E)** The potentiostatic polarization curve of PLM-2. Inset is the corresponding Nyquist curves before and after the DC test. **(F)** Illustration of the pore confinement effect in restricting the TFSI^−^ migration.

The ionic conductivity of the CPEs is an important factor for their application in batteries. The ionic conductivities and activation energies of the CPEs were evaluated by EIS measurements. Figure 2A showed the Nyquist plots of PEO-Li, PLM-1, PLM-2, and PLM-3 at 25°C. Each Nyquist plot was fitted into two parts (Dey et al., 2009): 1) the semicircle at the high-middle frequencies, which corresponded to ion conduction within the CPEs; 2) the linear part at the low frequencies, which was related to the ion blocking at the interface. The impedances were estimated from the intersection of the semicircular arc and the lateral axis. The ionic conductivities of PLM-1, PLM-2, and PLM-3 were calculated to be 1.01 × 10^−6^, 1.40 × 10^−6^, 7.97 × 10^−7^ S cm^−1^ at 25°C, which were over an order of magnitude higher than that of PEO-Li (4.98 × 10^−8^ S cm^−1^).

Since the addition of ZIF-67 could increase the amorphous degree of PEO matrix and thus the mobility of polymer chains, the densities of Li^+^ hopping sites could be significantly enhanced to achieve higher ionic conductivities. With the increase content of ZIF-67, the conductivity of PLM series first increased and then decreased. PLM-2 with an optimal ZIF-67 content of 10 wt% showed the highest ionic conductivity due to the rich hopping sites for Li^+^ transfer. In PLM-2, it was supposed that the Li^+^ could not only migrate through the amorphous areas in PEO matrix, but also via the high conductive pathways along the interface between ZIF-67 and PEO (Figure 2B). In PLM-3, ZIF-67 with much higher content tended to agglomerate, which then reduced the interfacial pathways and led to lower ionic conductivity. Figure 2C presented the temperature-dependent EIS curves and the corresponding Arrhenius plot of PLM-2. The ionic conductivity of PLM-2 was as high as 1.73 × 10^−4^ S cm^−1^ at 60°C, which was sufficient for utilization in batteries. The activation energy (E_a_) of CPEs was calculated according to the Arrhenius law (Wu and Guo, 2019). The E_a_ of PLM-2 was 0.87 eV, much lower than that of PEO-Li (Supplementary Figure S4, S5, S6 in the Supporting information). With the high ionic conductivity and low activation energy, PLM-2 showed great promise in achieving better electrochemical performance.

The electrochemical stability of SSEs is an important factor for their application in batteries. Thus, the electrochemical window of PLM-2 was measured via the linear sweep voltammetry (LSV) method by assembling a Li/PLM-2/stainless steel (SS) cell at 60°C. As shown in Figure 2D stable potential window up to 5.3 V was observed for PLM-2, much higher than that of PEO-Li. The excellent stability of PLM-2 could be attributed to the interaction between ZIF-67 and the ether oxygen atoms in PEO, which prevented the oxidation of PEO and considerably stabilized the PEO matrix at higher voltages. Besides, ZIF-67 had rich micropores and high surface area that could absorb the impurities and thus suppressed the possible side reaction within the PLM-2 electrolyte.

The Li_+_ transference number (t_+_) of PLM-2 was tested in a Li/PLM-2/Li symmetric cell using the Evans method (Evans et al., 1987; Xu D. et al., 2019; Xu W. et al., 2019; Wu and Guo, 2019). The calculated t_+_ for PLM-2 was about 0.41 (Figure 2E), which was much higher than that in PEO-Li (Supplementary Figure S7 and Supplementary Table S3 in the Supporting information). The improved t_+_ of PLM-2 could be ascribed to the confinement effect from ZIF-67. As illustrated in Figure 2F, ZIF-67 possessed cage-like micropores with the pore diameter and aperture of 1.16 and 0.34 nm, respectively.

The TFSI^−^ anions were long columnar with the size of 0.79 × 0.29 nm (Bai et al., 2018), which was slightly smaller than the pore aperture of ZIF-67. In PLM-2, the TFSI^−^ might firstly adjust their orientation and entered the ZIF-67 pores laterally via the lowest dimension. Then these TFSI^−^ would be confined in the pores with limited mobility. Comparatively, the Li^+^ cations with a much smaller size of around 0.076 nm were less affected and could mitigate through the MOFs’ lattice, thus leading to an increased t_+_. The high t_+_ of PLM-2 could effectively alleviate the formation of space charge area near the Li metal and favor a uniform Li deposition.

### 2.3 Cell performances

**FIGURE 3:**
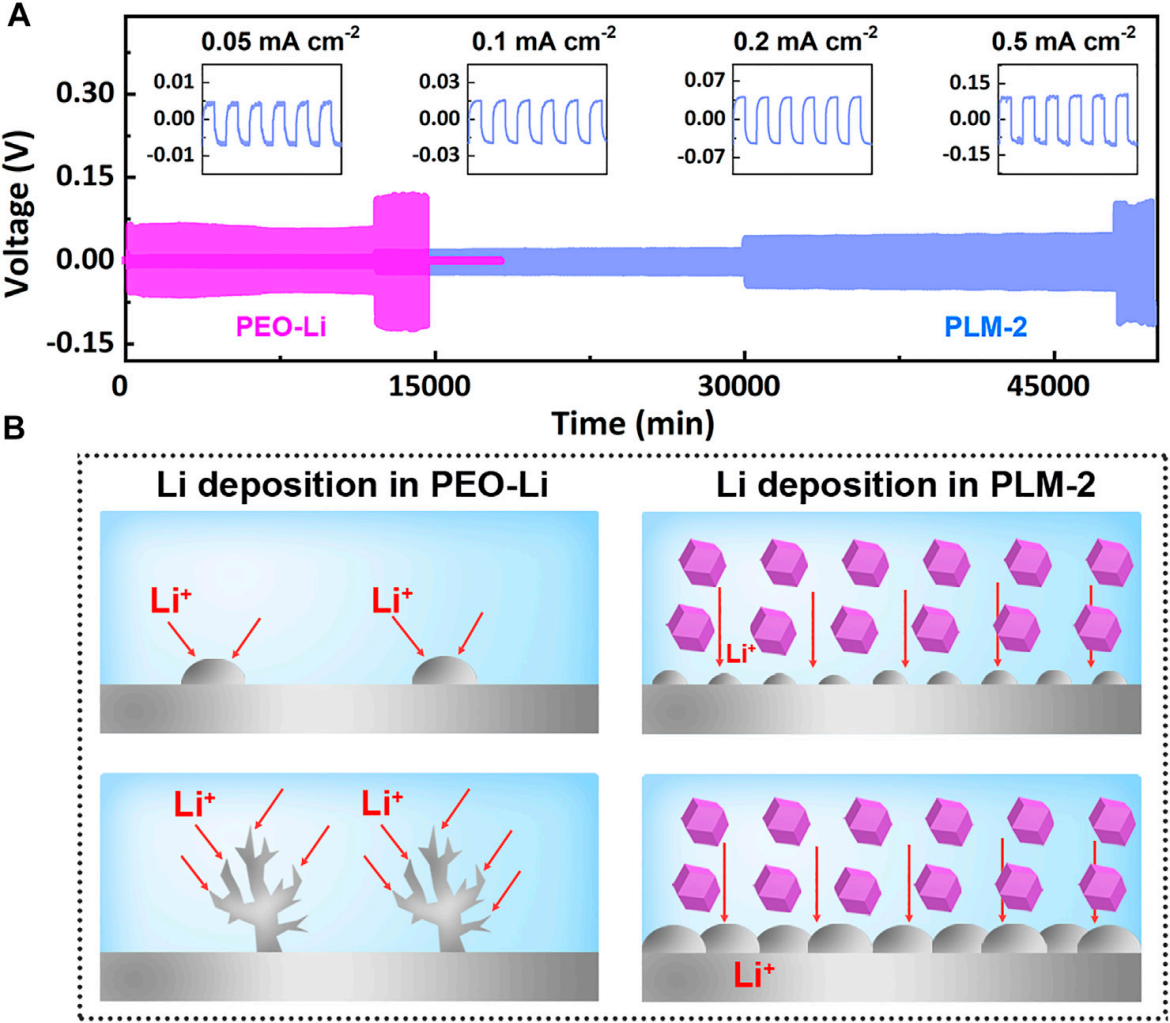
**(A)** The galvanostatic cycling of Li/PLM-2/Li symmetric cell and Li/PEO-Li/Li cell at 60°C. **(B)** Illustration of PLM-2 in suppressing Li dendrite formation.

The lithium asymmetric cell was assembled and cycled at 60°C at current densities of 0.05, 0.1, 0.2, and 0.5 mA cm^−2^ to evaluate the cycling stability of PLM-2 against Li metal. As showed in Figure 3A, the Li/PLM-2/Li cell presented a flat and small voltage polarization of 5, 15, 43, and 95 mV at 0.05, 0.1, 0.2, and 0.5 mA cm^−2^, respectively. The results suggested the high ionic conductivity and the superior interfacial compatibility between PLM-2 and Li metal upon Li plating and striping. In contrast, the Li symmetric cell using PEO-Li electrolyte exhibited much higher polarizations of 70 mV at 0.05 mA cm^−2^. The voltage further increased to 120 mV when the current density reached 0.1 mA cm^−2^, followed by a steep decline to near zero. The excellent stability of the symmetric cell could be ascribed to the following reasons: 1) the flexible PEO matrix with high-surface-area ZIF-67 could benefit the conformal and intimate contact between PLM-2 and Li metal, which reduced the interfacial impedance; 2) the ordered channels of ZIF-67 could provide well-defined pathways for the diffusion and uniform deposition of Li^+^, thus preventing the dendrite formation (Figure 3B); 3) ZIF-67 with abundant micropores could act as ion sieves that preferentially promoted Li^+^ transfer; 4) the rich porosity of ZIF-67 could trap the impurities, which effectively suppressed the side reaction and improved the cycling stability.

**FIGURE 4:**
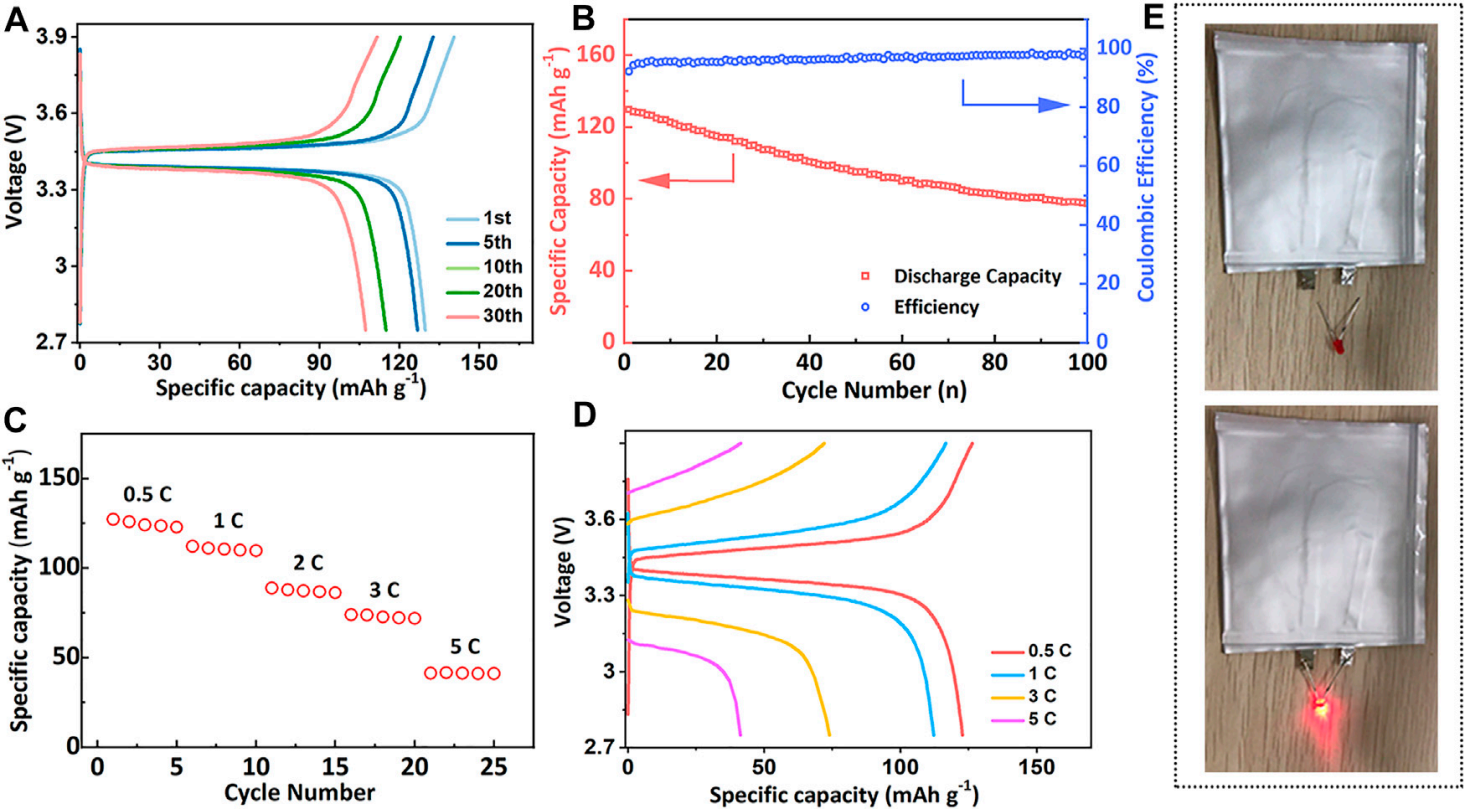
**(A)** The charge-discharge curves and **(B)** the cycling performance of the Li/PLM-2/LFP full cell at 0.2 C. **(C)** The rate performance and **(D)** the corresponding charge-discharge curves of the Li/PLM-2/LFP at 0.2 C. **(E)** The photos of lighted LED lamp by a PLM-2 based pouch cell.

The electrochemical performance of PLM-2 was further examined in the Li/PLM-2/LiFePO_4_ (LFP) full cell at 60°C. As illustrated in Figure 4A, the galvanostatic charging-discharging curves of the cell presented flat potential plateaus at 3.37 and 3.47 V at 0.2 C, indicating the small polarization of the cell.

The initial specific capacity reached 130 mAh g^−1^, followed by gradually reduction upon cycling. Capacities of 100 and 85 mAh g^−1^ were still maintained after 40 and 80 cycles, demonstrating a reasonable capacity retention (Figure 4B). The gradual decay of discharge capacity may be attributed to the following reasons: 1) the loss of LiFePO_4_ active materials due to the repetitive volume variation during battery cycling; 2) the deterioration of the interface between electrode and electrolyte that inhibited Li^+^ diffusion and intercalation. Figure 4C and Figure 4D showed the rate capability of the Li/PLM-2/LFP cell that was tested at 60°C. Capacities of 122, 112, and 75 mAh g^−1^ were obtained at 0.5, 1, 2, and 3C, respectively. The cell could even work normally at 5 C, highlighting the outstanding high-rate performance. The PLM-2 was also compatible with high-voltage cathode LiNi_0.5_Co_0.2_Mn_0.3_O_2_ (NCM). The Li/PLM-2/NCM cell delivered an acceptable capacity of 100 mAh g^−1^ after 30 cycles (Supplementary Figure S8 in the Supporting information). Additionally, a large-size pouch cell with high capacity was assembled, which successfully lighted up the light-emitting diode (LED) lamp at room temperature (Figure 4E). The excellent performance of the full cell could be attributed to the high ionic conductivity of PLM-2, the superior interfacial compatibility, and the stabilized interface during the charging/discharging process. The above results confirmed that PLM-2 as CPEs is promising to be applied in batteries for practical application.

### 2.4 Conclusion

In summary, a MOF-incorporated CPE (namely, PLM-2) was fabricated and utilized as electrolyte in SSBs. As solid fillers, ZIF-67 with high surface area and rich porosity enabled a sufficient contact and interaction with PEO matrix and LiTFSI, thus effectively improving the density of mobile Li^+^ and the conduction pathways. Accordingly, PLM-2 demonstrated a much higher ionic conductivity (1.40 × 10^−6^ S cm^−1^) than that of PEO-Li (4.98 × 10^−8^ S cm^−1^) at 25°C. In the Li symmetric cell, the polarization voltage was restricted within 0.1 V at various current densities over 800 h, verifying the superior interfacial compatibility and stability between PLM-2 and Li anode. The addition of ZIF-67 could guide the processes of Li stripping and plating, which effectively suppressed the Li dendrite formation. In the Li/PLM-2/LFP cell, a high initial capacity of 130 mAh g^−1^ and capacity retention of 85 mAh g^−1^ after 80 cycles were achieved at 0.2°C and 60°C. The excellent electrochemical performances, together with the facile preparation process, make PLM-2 promising for practical application. This work reveals the great prospect of MOF to be used as functional fillers to synthesize high-performance CPEs, expanding the application scope of MOFs in SSBs.

## 3 Experimental section

### 3.1 Materials synthesis

ZIF-67 was synthesized according to the previous works (Xia et al., 2014). Briefly speaking, 1.4 g Co(NO_3_)_2_⋅6H_2_O and 3.2 g 2-methyimidazole were each dissolved in 100 ml methanol at room temperature. The above salt solutions were mixed, stirred for 20 min, and then kept for 24 h. The as-prepared samples were then filtered out, washed and vacuum dried.

The CPEs, including PLM-1, PLM-2 and PLM-3, were prepared at room temperature using the solution cast method with different concentrations of ZIF-67. For the synthesis of PLM-1, 2.4 g PEO and 0.3 g LiTFSI were dissolved in acetonitrile in a glass container. The mixture was magnetically stirred until a homogeneous solution was formed. Then 0.1 g ZIF-67 was added to the above solution and stirred for 12 h. The slurry was cast onto a horizontal Teflon plate and dried in a vacuum oven at 50°C for 24 h to completely remove the acetonitrile solvent. A similar process was adopted for the preparation of PLM-2 and PLM-3. The adding weight of ZIF-67 was changed to 0.3 and 0.5 g for PLM-2 and PLM-3, respectively. The other parameters, including the weight of PEO and LiTFSI, the forming and drying procedures remained the same as those for PLM-1. The thickness of CPEs membrane was 125 μm. All the procedures were performed in an argon-filled glove box.

### 3.2 Characterization

Powder X-ray diffraction (PXRD) was performed on a Rigaku SmartLab 9 kW diffractometer with a copper target (*λ* = 1.5406 Å). Thermal gravimetric analysis (TGA) was recorded on a Mettler Toledo TGA/DSC 3 + analyzer under nitrogen flow at a heating rate of 2°C min^−1^ from 30 to 600°C. Differential Scanning Calorimetry (DSC) analysis was conducted on a NETZSCH DSC 214 instrument with a heating/cooling rate of 10 ^°^C/min from -100 to 100°C. Nitrogen sorption isotherms were measured at 77 K on a Quantachrome Autosorb-IQ gas adsorption analyzer. Prior to the sorption studies, the samples were degassed under dynamic vacuum. Using the adsorption isotherms, the specific surface areas were calculated by Brunauer–Emmett–Teller (BET) method. The pore size distributions were obtained using the quenched solid density functional theory (QSDFT) method.

### 3.3 Electrochemical measurement

The electrochemical measurements were carried out on an electrochemical workstation (Solartron Analytical 1,400 or Autolab M204) and Landian multichannel battery tester. The ionic conductivity of the CPEs were obtained from the electrochemical impedance spectroscopy (EIS). The frequency range was from 1 Hz to 1 M Hz, and the disturbance voltage was 10–50 mV. Before the testing, the corresponding cells were assembled using two polished stainless-steel blocking electrodes, between which the as-prepared CPE membranes were clamped. The ionic conductivity σ (S cm^−1^) was calculated from Equation 1:

σ=d/(S×R) (1)

Here, d (cm), S (cm^2^), and R (Ω) are the thickness, surface area, and resistance of the CPE membranes, respectively. The electrochemical stability window of the CPEs was measured via linear sweep voltammetry (LSV) at room temperature, where the stainless-steel and a lithium metal were used as working electrode and reference electrode, respectively. The voltage range was from 2 to 6 V with a scan rate of 0.2 mV s^−1^. The Li^+^ transference number (t_+_) of the CPEs were obtained by the chronoamperometry test and EIS tests in a symmetric Li/CPEs/Li cell. The t_+_ of the CPEs was calculated based on Equation 2:

$$t_{+}=\left[I_{s}\left(\Delta V-I_{0}R_{0}\right)\right]/\left[I_{0}\left(\Delta V-I_{s}R_{s}\right)\right]$$ (2)

Here, ΔV (V) is the potential applied. I_0_ (A) and I_s_ (A) are the initial and steady-state currents in the chronoamperometric curve. R_s_ (Ω) and R_0_ (Ω) are the initial and steady-state resistances obtained from the EIS curves.

To assemble the symmetric Li/CPEs/Li cells, the CPEs were sandwiched between two pieces of Li metal discs and sealed within a coin-type cell. Galvanostatic cycling was conducted on the as-obtained symmetric Li/CPEs/Li cells at different current densities (0.05, 0.1, 0.2, 0.5 mA cm^−2^) at 60°C. A Li/CPEs/LiFePO_4_ full cell was assembled to evaluate the capacity and cycling stability. The cathode was prepared via a blade-coating method and was consisted of LiFePO_4_ (LFP), super P, PVDF, PEO and LiTFSI. The LFP accounted for 80 wt% of the cathode. The loading of LFP was about 1 mg in the Li/PLM-2/LFP cell. The full cell was cycling between 2.75 and 3.9 V at 60°C. The galvanostatic charge-discharge method was conducted at 0.2 C (1 C = 140 mA g^−1^)). The preparations of samples and assembly of cells were performed in a glove box under an argon atmosphere.

### 3.4 Materials

LiTFSI (99.9%) was purchased from Aldrich. 2-Methylimidazole (98%), Polyethylene oxide (PEO, Mw: 300,000) and polyacrylonitrile (PAN, Mw: 150,000) were purchased from Macklin. Co(NO_3_)_2_·6H_2_O (97.7%) was purchased from Alfa Aesar.

## Data Availability

The raw data supporting the conclusions of this article will be made available by the authors, without undue reservation.
