# Supplementary material for: Metal-organic framework (MOF)-incorporated polymeric electrolyte realizing fast lithium-ion transportation with high Li+ transference number for solid-state batteries
Source: Front Chem. 2022 Oct 3;10:1013965. doi: 10.3389/fchem.2022.1013965 (PMC9574007; doi:10.3389/fchem.2022.1013965)
Supplement: Supplementary file 1 [file DataSheet1.docx]

Metal-organic framework (MOF)-incorporated polymeric electrolyte realizing fast lithium-ion transportation with high Li^+^ transference number for solid-state batteries

Yifan Xu^2^, Ruo Zhao^1*^, Jianjun Fang^2^, Zibin Liang^3^, Lei Gao^3^, Juncao Bian^2^, Jinlong Zhu^2^, Yusheng Zhao^2^

^1^Institute for Advanced Study Shenzhen University, Shenzhen, China.

^2^Shenzhen Key Laboratory of Solid State Batteries, Guangdong Provincial Key Laboratory of Energy Materials for Electric Power, Guangdong-Hong Kong-Macao Joint Laboratory for Photonic-Thermal-Electrical Energy Materials and Devices, Academy for Advanced Interdisciplinary Studies, Southern University of Science and Technology, Shenzhen, China.

^3^School of Materials Science and Engineering Peking University Beijing, China.

*** Correspondence:**Ruo Zhao
zhaor@sustech.edu.cn

Supplementary Material


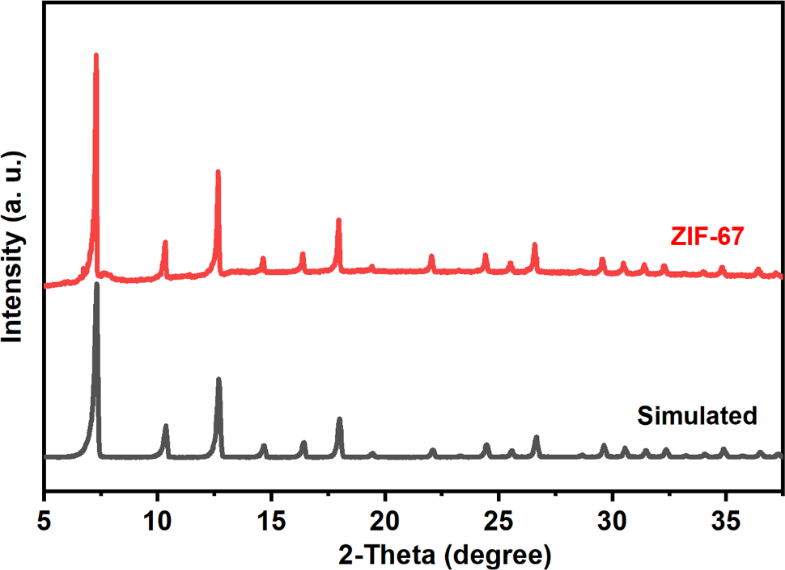


**Figure S1.** XRD patterns of the as-synthesized ZIF-67 and simulated one.


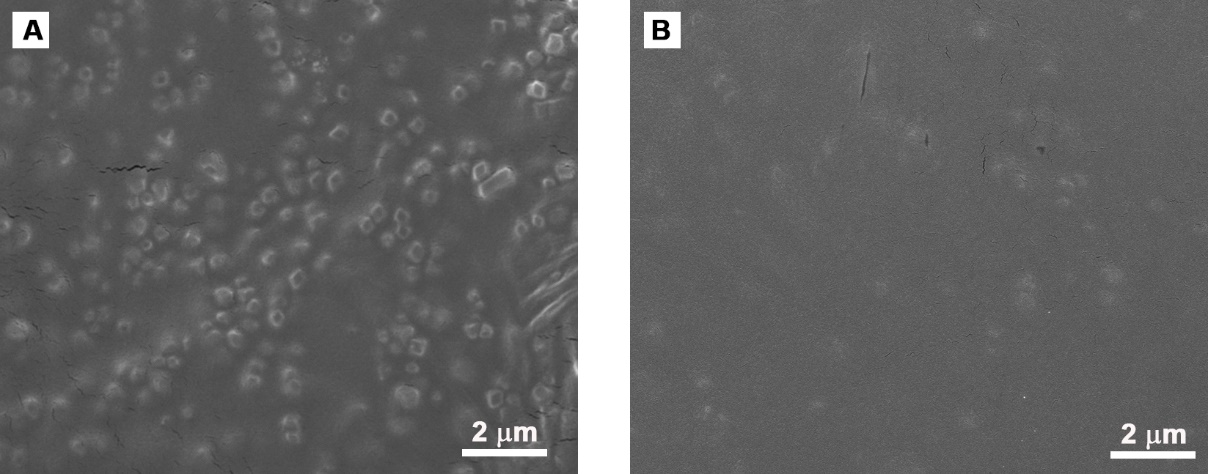


**Figure S2.** (A) The SEM image of PLM-3, illustrating the phenomenon of particle agglomeration. (B) The SEM image of PLM-1, showing the sparse distribution of ZIF-67 nanoparticles.


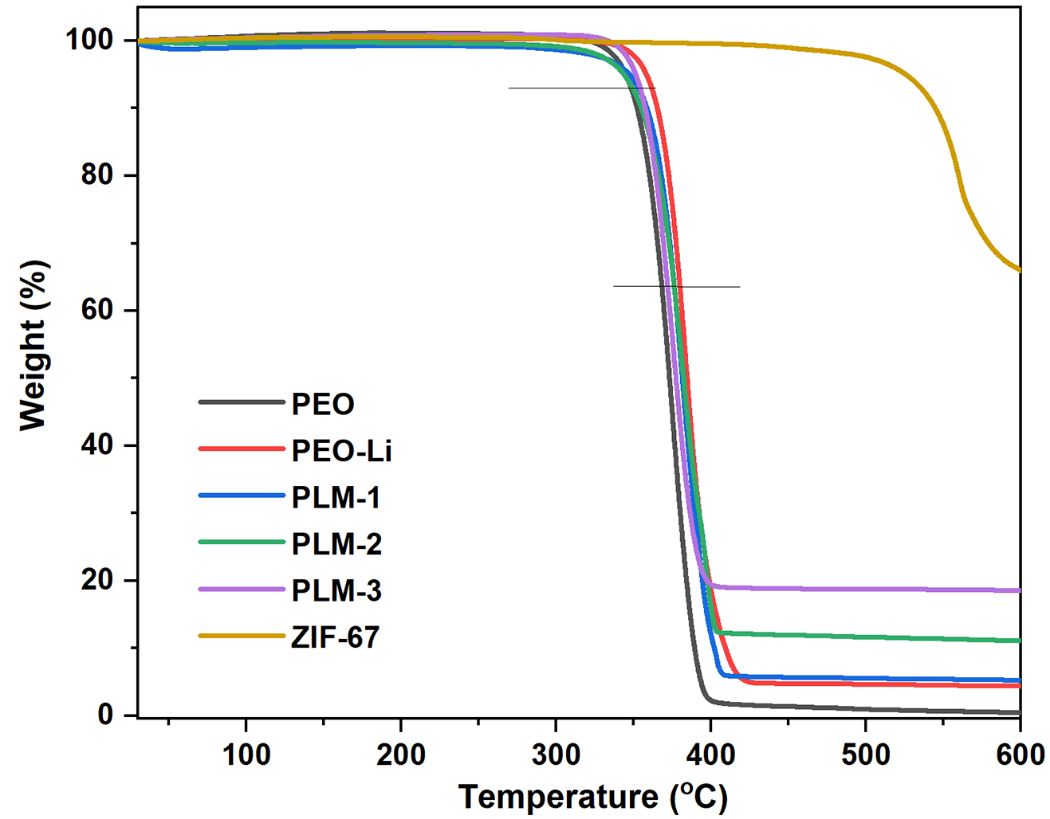


**Figure S3.** The TGA curves of PEO, PEO-Li, PLM-1, PLM-2, PLM-3 and ZIF-67.


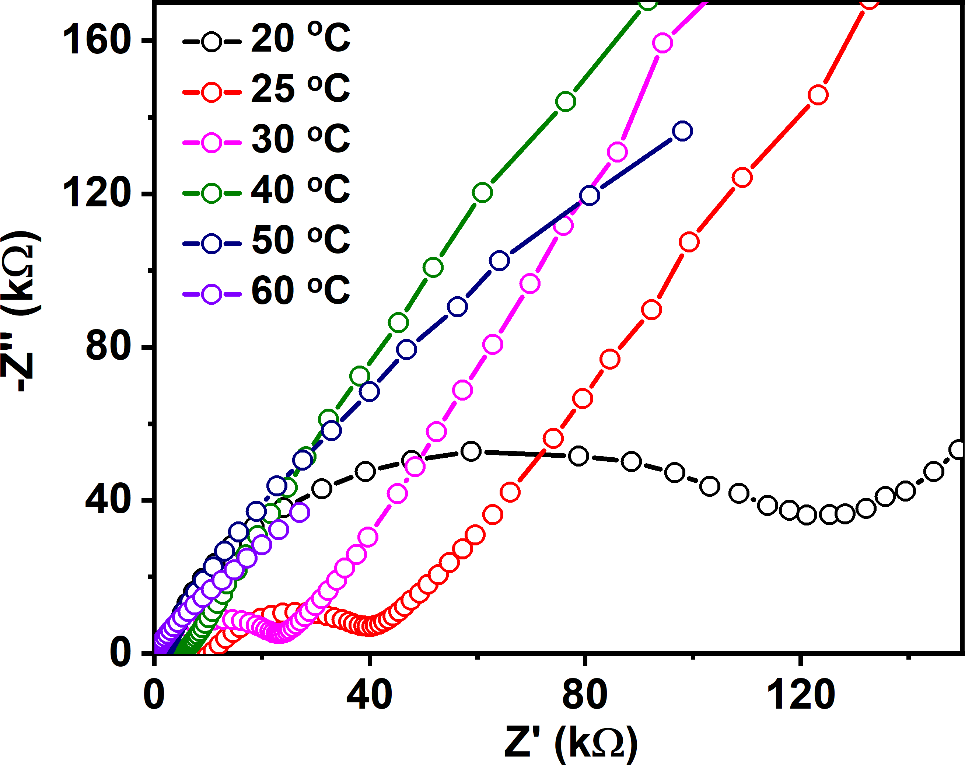


**Figure S4.** The Nyquist plots for PEO-Li at various temperatures


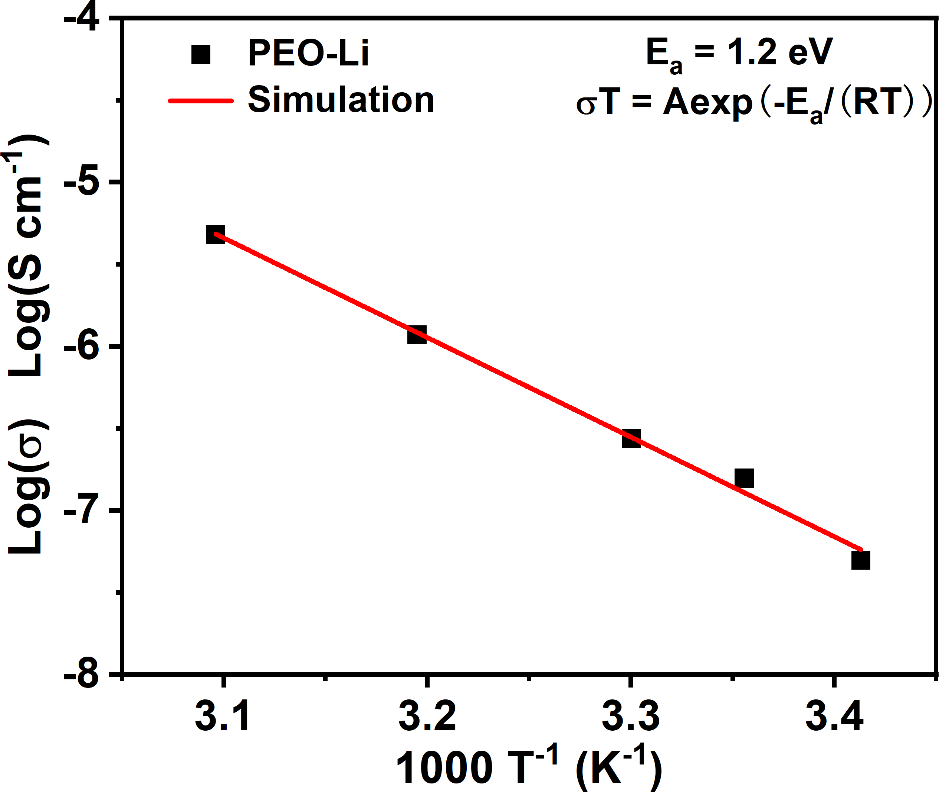


**Figure S5.** The Arrhenius plot of the PEO-Li and the corresponding linear fitting.


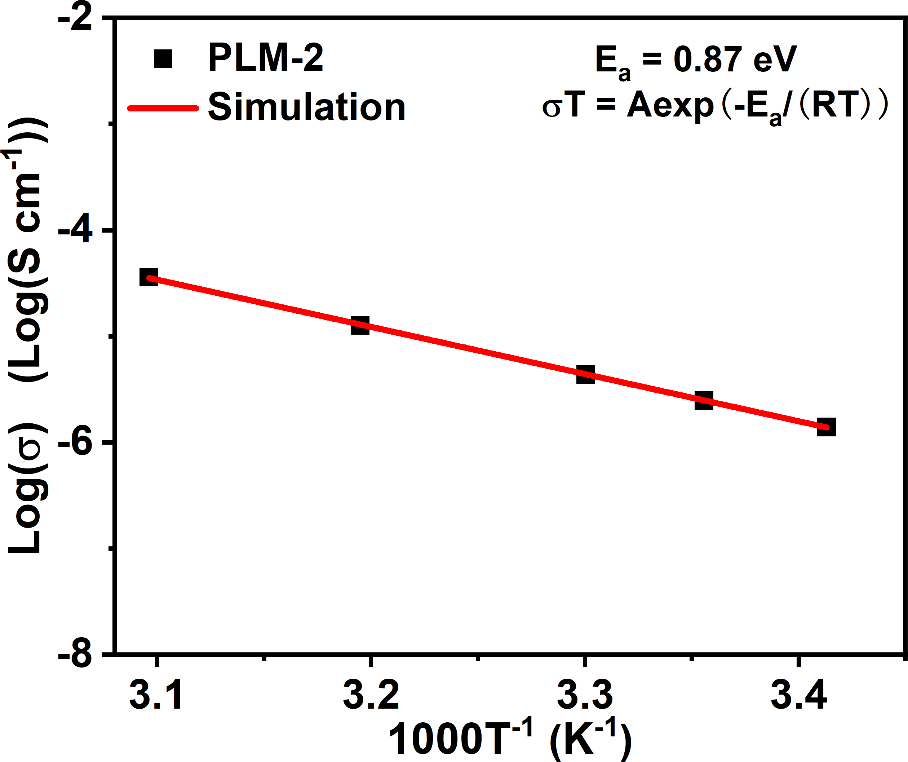


**Figure S6.** The Arrhenius plot of the PLM-2 and the corresponding linear fitting.


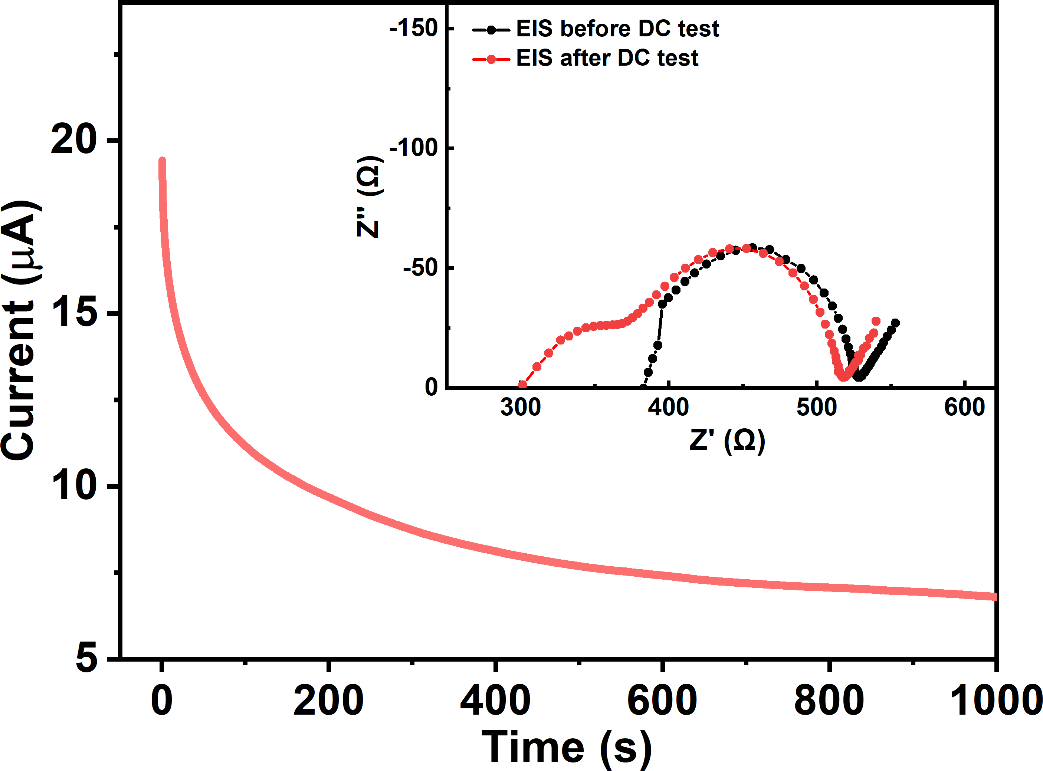


**Figure S7.** The potentiostatic polarization curve of PEO-Li. Inset is the corresponding Nyquist curves before and after the DC test. The lithium ion transfer number of PEO-Li is equal to 0.29.


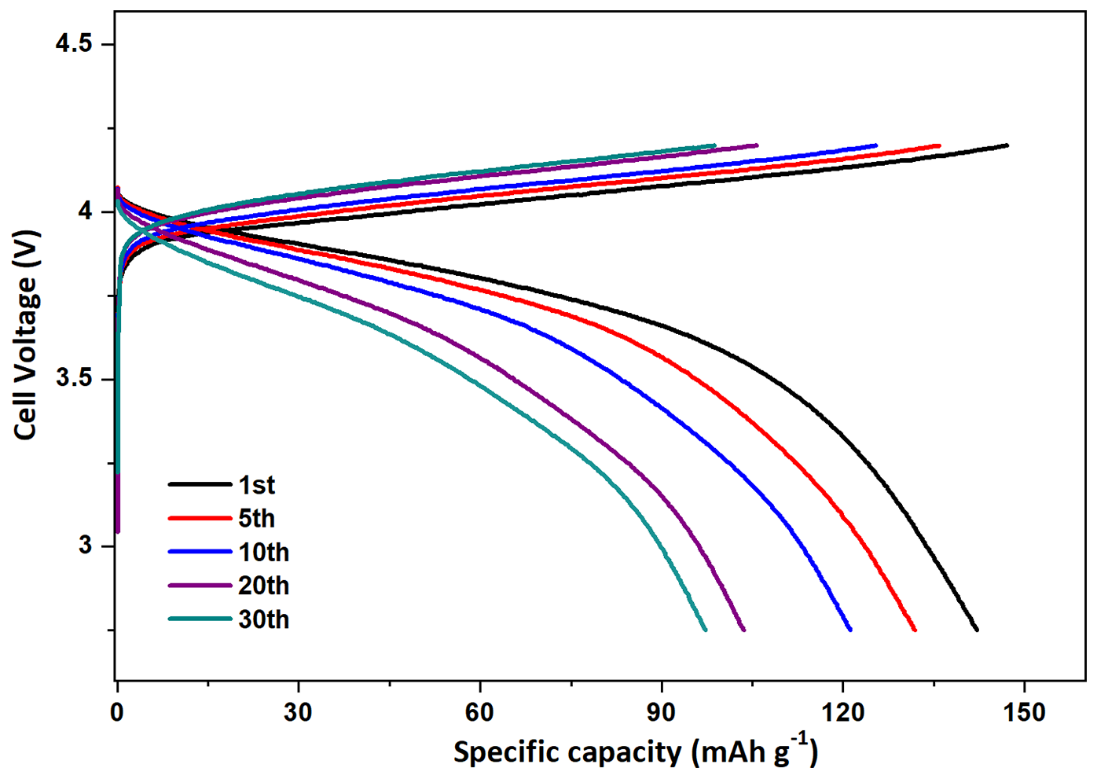


**Figure S8.** The charge-discharge curves of the Li/PLM-2/LiNi_0.5_Co_0.2_Mn_0.3_O_2_ full cell at 0.2 C and 60 ^o^C.


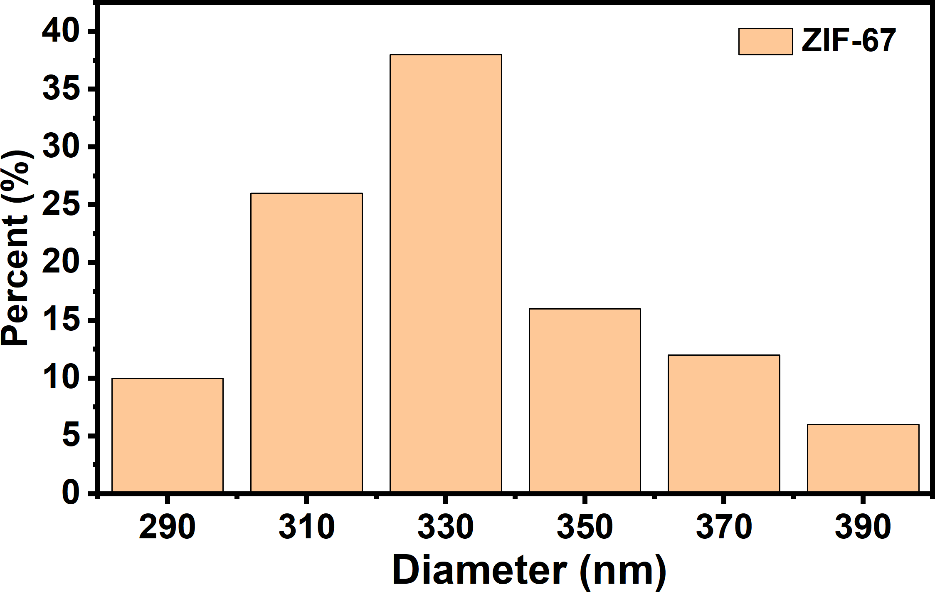


**Figure S9.** Diameter distribution of ZIF-67.


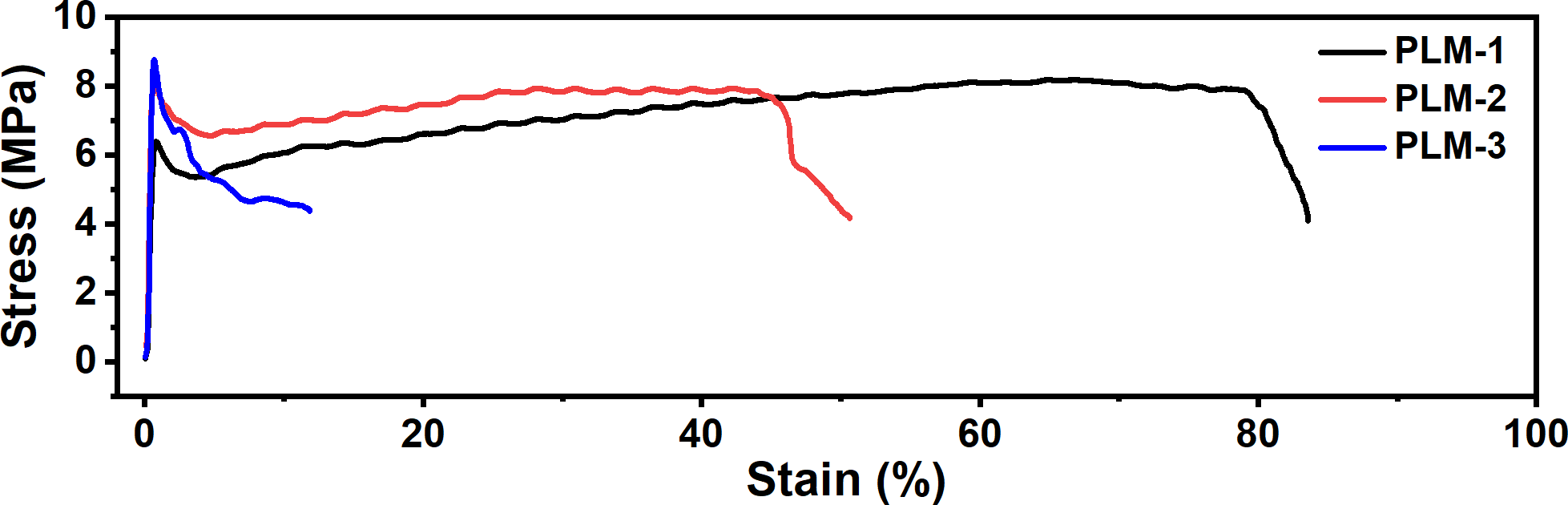


**Figure S10** Mechanical strength of PLM-1,PLM-2 and PLM-3.


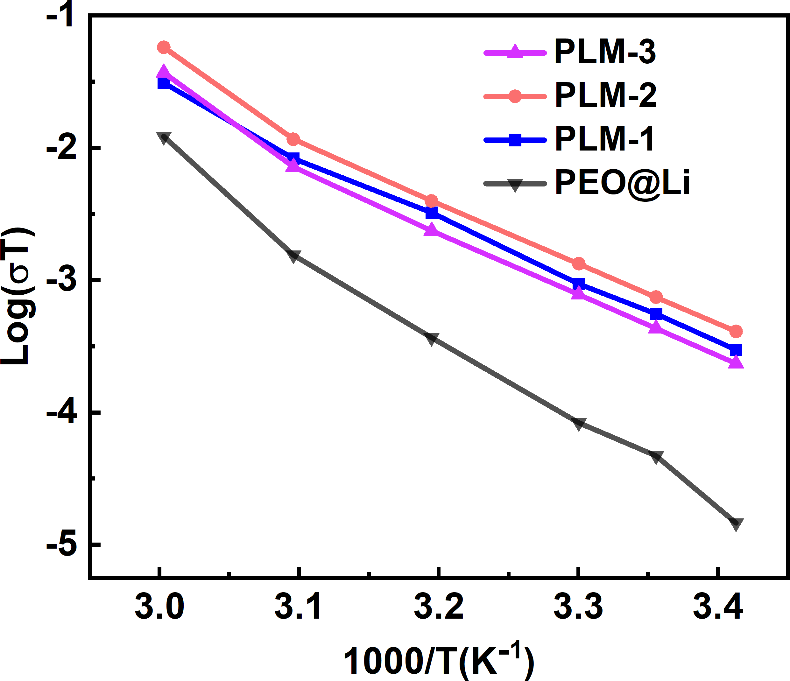


**Figure S11** EIS curves of PLM-3, PLM-2, PLM-1 and PEO-Li.


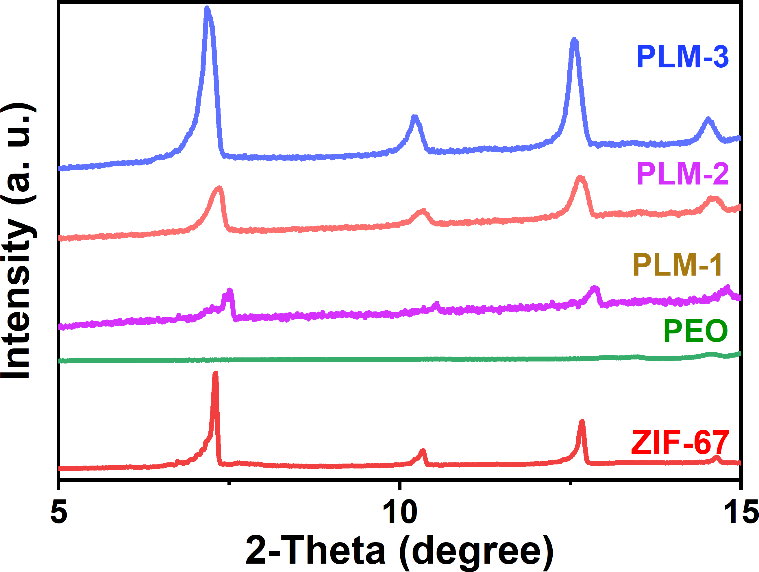


**Figure S12** XRD patterns of the as-synthesized ZIF-67, PEO, PLM-1, PLM-2 and PLM-3.

**Table S1.** The summary of melting temperature (T_m_), melting heat (ΔH_m_) and crystallinity degree (χ_c_) of PEO, PEO-Li, PLM-1, PLM-2, and PLM-3.

| Sample | T_m_ | ΔH_m_ (J/g) | χ_c_ |
| --- | --- | --- | --- |
| PEO | 70 | 193.2 | 100 % |
| PEO-Li | 69 | 128.4 | 66 % |
| PLM-1 | 70 | 126.3 | 65 % |
| PLM-2 | 66 | 66.2 | 34 % |
| PLM-3 | 67 | 89.6 | 46 % |

**Table S2.** Calculation process of PLM-2 activation energy.

Calculation procedure: (1) measuring the ionic conductivities of CPEs at different temperatures; (2) plotting figures using 1000/T as the X-axis and Log (σ) as the Y-axis; (3) obtaining the slop by a linear fitting method; (4) calculating the activation energy using E_a_ = -2300[(Log(S_2_)-Log(S_1_))]/[(1000/T_2_)-(1000/T_1_)]=2300k/R, For PLM-2,Ea=0.87 eV, The slope of k is 1000/T as the X-axis and Log (σ) as the Y-axis.

| Temperature (K) | R (Ω) | 1000/T (K^-1^) | σ (S cm^-1^) | Log (σ) (Log (S cm^-1^)) |
| --- | --- | --- | --- | --- |
| 293K | 4455 | 3.412 | 1.40 X 10^-6^ | -5.85 |
| 298K | 2500 | 3.355 | 2.49 X 10^-6^ | -5.60 |
| 303K | 1421 | 3.300 | 4.38 X 10^-6^ | -5.35 |
| 313K | 491 | 3.194 | 1.27 X 10^-5^ | -4.89 |
| 323K | 173 | 3.095 | 3.60 X 10^-5^ | -4.44 |

**Table S3.** Calculation process of lithium ion transfer number in PEO-Li and PLM-2.

Calculation procedure: (1) EIS tests in a symmetric Li/CPEs/Li cell (2) chronoamperometry test in a symmetric Li/CPEs/Li cell. (3) EIS tests in a symmetric Li/CPEs/Li cell. (4) t_+_ = [I_s_(ΔV-I_0_R_0_)]/ [I_0_(ΔV-I_s_R_s_)], Here, ΔV (V,0.01V) is the potential applied. I_0_ (A) and I_s_ (A) are the initial and steady-state currents in the chronoamperometric curve. R_s_ (Ω) and R_0_ (Ω) are the initial and steady-state resistances obtained from the EIS curves.

| Sample (^o^C) | R_o_ (Ω) | R_s_ (Ω) | I_o_ (uA) | I_s_ ( uA ) | t_+_ |
| --- | --- | --- | --- | --- | --- |
| PEO-Li | 150 | 215 | 19.45 | 6.8 | 0.29 |
| PLM-2 | 202 | 215 | 19.3 | 10.2 | 0.41 |
